# High clustering rate and genotypic drug-susceptibility screening for the newly recommended anti-tuberculosis drugs among global extensively drug-resistant *Mycobacterium tuberculosis* isolates

**DOI:** 10.1080/22221751.2022.2099304

**Published:** 2022-07-27

**Authors:** Kanwara Trisakul, Ditthawat Nonghanphithak, Pratchakan Chaiyachat, Orawee Kaewprasert, Kankanon Sakmongkoljit, Wipa Reechaipichitkul, Angkana Chaiprasert, David Blair, Taane G. Clark, Kiatichai Faksri

**Affiliations:** a Department of Microbiology, Faculty of Medicine, Khon Kaen University, Khon Kaen, Thailand; b Research and Diagnostic Center for Emerging Infectious Diseases (RCEID), Khon Kaen University, Khon Kaen, Thailand; c Department of Geotechnology, Faculty of Technology, Khon Kaen University, Khon Kaen, Thailand; d Office for Research and Development, Faculty of Medicine Siriraj Hospital, Mahidol University, Bangkok, Thailand; e James Cook University, Townsville, Australia; f London School of Hygiene and Tropical Medicine, London, UK

**Keywords:** XDR-TB, bedaquiline, clofazimine, linezolid, delamanid, pretomanid, cycloserine

## Abstract

Multidrug-resistant tuberculosis (MDR-TB) and extensively drug-resistant tuberculosis (XDR-TB) make TB difficult to control. Global susceptibility data for six newly recommended anti-TB drugs against M/XDR-TB are still limited. Using publicly available whole-genome sequences, we determined the proportion of 513 phenotypically XDR-TB isolates that carried mutations associated with resistance against these drugs (bedaquiline, clofazimine, linezolid, delamanid, pretomanid and cycloserine). Mutations of *Rv0678* and *Rv1979c* were detected in 69/513 isolates (13.5%) for bedaquiline resistance and 79/513 isolates (15.4%) for clofazimine resistance with additional *mmpL5* mutations. Mutations conferring resistance to delamanid were detected in *fbiB* and *ddn* genes for 11/513 isolates (2.1%). For pretomanid, a mutation was detected in the *ddn* gene for 3/513 isolates (0.6%). Nineteen mutations of *pykA, cycA, ald*, and *alr* genes, conferring resistance to cycloserine, were found in 153/513 isolates (29.8%). No known mutations associated with linezolid resistance were detected. Cluster analysis showed that 408/513 isolates fell within 99 clusters and that 354 of these isolates were possible primary drug-resistant TB (292 XDR-TB, 57 pre-XDR-TB and 5 MDR-TB). Clonal transmission of primary XDR isolates might contribute significantly to the high prevalence of DR-TB globally.

## Introduction

Tuberculosis (TB) is an infectious disease caused by *Mycobacterium tuberculosis* (*Mtb*). An estimated 9.9 million people became infected in 2020, potentially leading to 1.3 million deaths among HIV-negative people [1]. Drug-resistant (DR) TB has a relatively high mortality rate. In 2020, 132,222 cases of multidrug-resistant TB (MDR-TB; resistant to isoniazid and rifampicin) or rifampicin-resistant TB (RR-TB) were detected globally. Additionally, 25,681 cases of pre-extensively drug-resistant TB (pre-XDR-TB; resistant to rifampicin and any fluoroquinolone (FQ)) or XDR-TB (resistant to rifampicin, plus any FQ, plus at least one of bedaquiline (BDQ) and linezolid (LZD)), were reported [1]. Treatment of these serious forms of DR-TB is very challenging. Recently, the World Health Organization (WHO) released recommendations for treatment of DR-TB, especially XDR-TB [2]. The six newly recommended drugs are BDQ, LZD, clofazimine (CFZ), delamanid (DLM), pretomanid (PA-824 or PA) and cycloserine (CS). These drugs currently form the last line of defense against DR-TB, especially XDR-TB. However, there is as yet no information available for the extent to which existing TB isolates may already exhibit resistance against these newly recommended drugs.

Finding the appropriate treatment regimen for XDR-TB is challenging. There is only limited information concerning the efficacy of these six newly recommended drugs against actual XDR-TB [3]. XDR-TB isolates are more likely to exhibit resistance due to their exposure to drugs during the treatment period, and we therefore focused on such isolates. There are two classes of methods for screening TB isolates for DR. The older class is phenotypic drug-susceptibility testing (DST), which is the gold-standard method. Genotypic susceptibility testing is becoming more common but depends on the availability of whole-genome data and of a database of mutations (including single-nucleotide polymorphisms (SNPs) and insertions/deletions (indels)) that are known to confer resistance to particular drugs. Although phenotypic DSTs are currently used in routine laboratory work-up, it is difficult to collect sufficient XDR samples to test for phenotypic DST because of the low numbers of cases. Therefore, obtaining an inventory of XDR-phenotype strains to study for resistance against crucial anti-TB drugs is also very difficult. Hence, there is no data relating to the frequency of phenotypic and/or genotypic resistance against these important drugs available as yet. It is easier to access WGS-defined data for XDR samples from public sources, although the value of these might be limited by incomplete metadata and biased through convenience sampling. Nevertheless, genotypic methods are currently the most effective way to estimate DR-TB prevalence by detecting mutations conferring resistance against these newly recommended drugs, which is essential for classifying isolates as pre-XDR-TB or M/XDR-TB. Consequences of this information for disease control and patient management are obvious [4].

Controlling widespread TB outbreaks requires tracking recent transmission. In addition to information about likely drug resistance, WGS data can also provide information on clustering and timing of transmission chains, in which strains are clustered based on their genotypic similarities [5]. One previous study reported a high level of transmission of MDR-TB and XDR-TB clusters across four Thai provinces [6]. Another study revealed clustering patterns and possible clonal transmission of MDR-TB and XDR-TB in Thailand using WGS data [7]. However, the extent of expansion of XDR-TB clusters and possible clonal transmission events globally are still unknown. In this study, we aimed, by using WGS data to: (1) to determine the proportion of phenotypic XDR strains collected from the global XDR-TB strain collection that were also genotypically DR against six new drugs (BDQ, CFZ, LZD, DLM, PA-824 and CS) [7–15] and (2) to investigate whether the genetic diversity in XDR-TB strains revealed clustering and possible clonal transmission events.

## Materials and methods

### Study population

From global public databases, we retrieved Illumina WGS fastq datasets of 535 *Mtb* isolates that were XDR-TB (according to phenotypic DSTs) [7–15]. The (older) definition of XDR-TB employed was that such strains are resistant to any FQ and at least one of three injectable second-line drugs (capreomycin (CM), kanamycin (KM), and amikacin (AM)), in addition to isoniazid and rifampicin [16]. Genotypic DSTs and lineage classification of the *Mtb* strains were determined using the online tool, TB-Profiler version 2.8.6 [17]. We also retrieved data on the geographical origin of each isolate. In addition, we used 18 serially collected paired isolates from nine patients [7] as internal controls for determining SNP-distance cut-off values to identify clusters of isolates. We also retrieved Illumina WGS fastq datasets for 23 pan-susceptible (panS) *Mtb* isolates [4] to determine mutation-frequency cut-off values that allowed us to identify neutral mutations (mutations found in more than 5% of the panS population). The study protocol was approved by the Center for Ethics in Human Research, Khon Kaen University (HE601249).

### Assembly of a catalog of known mutations associated with resistance to six new drugs

We assembled a catalog of the known mutations associated with resistance to the six new drugs. Information from well-known mutation databases such as TB-Profiler, PubMed, and WHO were included (Supplementary File 1: Table S1). Genomic positions of the known mutations were calculated using the Mycobrowser [18] and PhyTB [19].

Our final list included 367 known mutations associated with resistance to the six new drugs including 154 mutations associated with BDQ resistance, 36 mutations associated with CFZ resistance, 68 mutations associated with DLM resistance, 29 mutations associated with PA-824 resistance, 63 mutations associated with CS resistance, and 17 mutations associated with LZD resistance (Supplementary File 2: Table S2). These mutations were used in this study.

### Whole-genome sequencing analysis

The quality of the WGS data (Illumina platform) was checked using FastQC version 0.11.7 [20]. Low-quality data were filtered using Trimmomatic version 0.38 [21]. Then, short-read DNA sequences were mapped to the H37Rv reference genome (NC_000962.3) using BWA-MEM version 0.7.12 [22]. SAM was converted to BAM format and BAM file sorted using SAMtools version 0.1.19 [23]. We used GATK version 3.6.0 [24] in the local realignment process. We used BCFtools version 1.9 [25] and GATK version 3.6.0 [24] to call variants with mapping quality of at least 50 and base alignment quality (Q score) of 20, focusing on SNPs and indels. The called variants were filtered with 10-fold minimum depth of coverage and Q score of 20. The intersecting variants from SAMtools and GATK were further analysed. We created mpile up files using SAMtools version 0.1.19 [23] and created the coverage files from mpile up files. The SAMtools mpileup ﬁles and VCF ﬁles were used to generate the combined nucleotide frequencies among strains at each SNP position. The mutation frequency for each drug was extracted from the analysed data using an in-house Python script. The SNP positions detected using TB-Profiler [17] were also included to increase the sensitivity.

### Genotypic drug-susceptibility test for six drugs

The combined variant positions (n = 25,062) among strains in the studied population (n = 513) were analysed. Based on the mutations from our combined catalog of DR-associated mutations (Supplementary File 1: Table S1), the isolates carrying mutations associated with resistance against each of the six drugs were identified. All 513 phenotypic XDR-TB strains were further classified as genotypic XDR-TB, pre-XDR-TB or MDR-TB (Supplementary File 3: Table S3).

### Phylogenetic analysis

The combined variants (n = 57,788) among the 513 isolates (plus *M. canettii* CIPT 140070010 as the outgroup) were analysed. This variant set was filtered with ≥5-fold coverage and ≥0.75 frequency. We then excluded known mutations associated with DR-TB (179 SNPs) shared among strains and SNPs found only relative to *M. canettii* (32,726 SNPs). A maximum-likelihood phylogenetic tree based on 17,988 high-confidence SNPs was inferred using RAxML version v8.2.10 [26] with 1,000 bootstrap replicates and a general time-reversible (GTR) with gamma-distribution model. Visualization of the phylogenetic tree was done using iTOL [27].

### Cluster analysis

We defined members of a cluster as differing from one another by ≤11 SNPs. We analysed pairwise SNP distances within each cluster using snp-dists version 0.7.0 [28]. The clustering percentage was calculated by (no. clustered isolates/ total no. isolates) × 100. Possible primary DR-TB was differentiated from acquired DR-TB based on acquisition of additional resistance-associated mutations, especially those associated with resistance to FQ, KM or CM, drugs that are used for classification of DR-TB [7] (based on the former definitions of pre-XDR-TB and XDR-TB). Although pre-XDR-TB and XDR-TB might be regarded as subgroups of MDR-TB, we have treated all three DR-TB categories as separate groups. Phylo-maps were built using ArcGIS software (version 10.5).

We analysed the association between DST profile vs cluster and DST profile vs possible primary DR-TB transmission clusters using χ^2^ tests or Fisher’s exact test. *P* values < .05 were considered statistically significant.

## Results

### Study population

**Figure 1.**
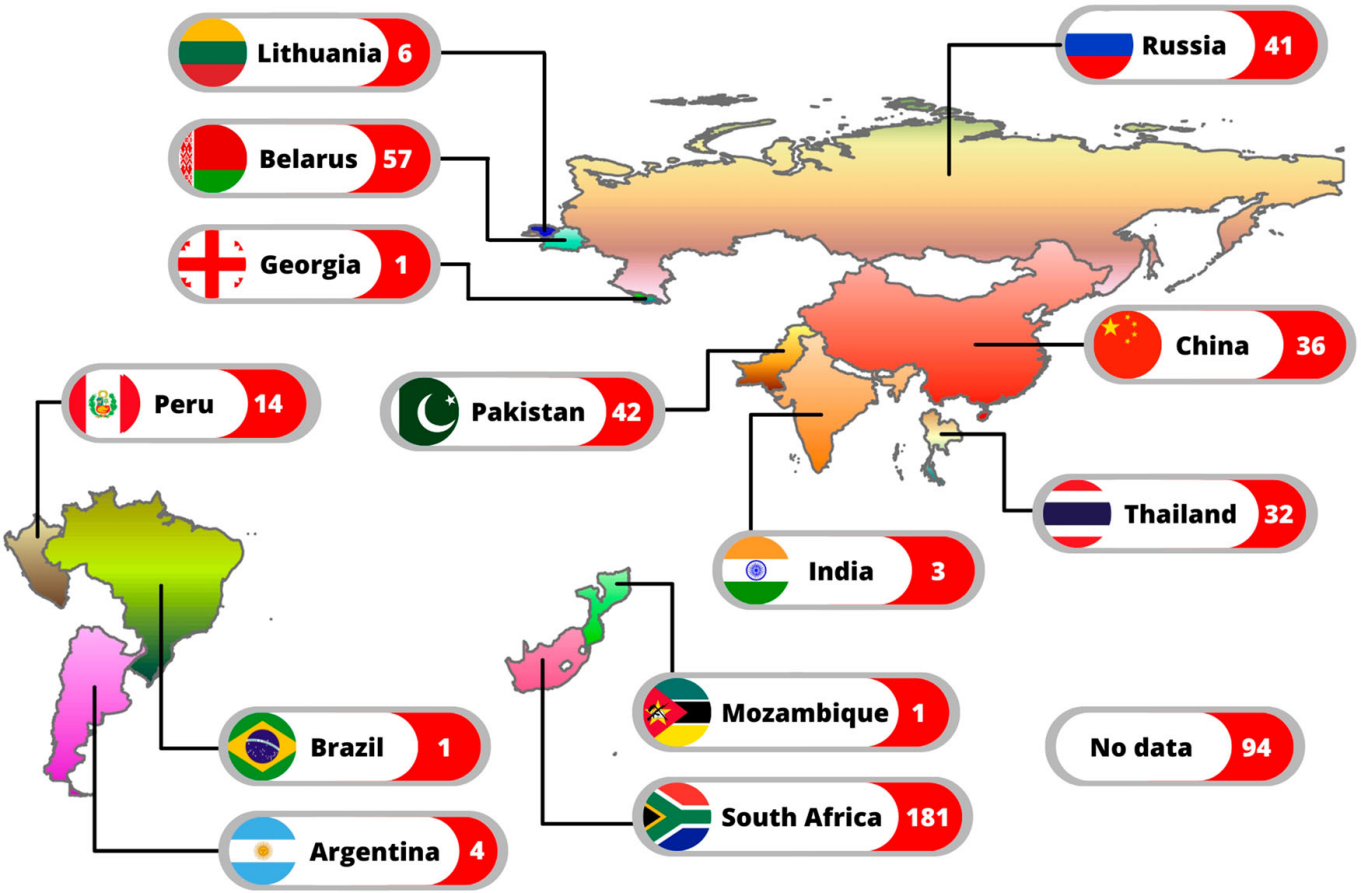
Countries of origin of 513 phenotypic XDR isolates. Most were from South Africa (n = 181: 35.3%), Belarus (n = 57: 11.1%) and Pakistan (n = 42: 8.2%). There was no geographical data available for some isolates (n = 94: 18.3%).

WGS data for 535 phenotypically XDR-TB isolates were retrieved from public sources. These isolates originated from at least 13 countries. TB-Profiler identified them as genotypically XDR-TB (n = 402: 75.1%), pre-XDR-TB (n = 94: 17.6%), MDR-TB (n = 17: 3.2%), other drug-resistant (n = 13: 2.4%), susceptible (n = 8: 1.5%) and incomplete data (n = 1: 0.2%). Only the 513 isolates identified genotypically as XDR-TB, pre-XDR-TB and MDR-TB were included in this study (Supplementary File 3: Table S3). Where known, the distribution of the strains among different countries is shown (Figure 1). The three countries contributing the most strains were South Africa (n = 181: 35.3%), Belarus (n = 57: 11.1%) and Pakistan (n = 42: 8.2%). Geographical data were unavailable for some isolates (n = 94: 18.3%).

### Proportions of studied isolates genotypically resistant against six drugs

We determined the proportions of isolates resistant against each of the six drugs according to the mutations they carried. Mutations in *Rv0678* and *Rv1979c* associated with BDQ resistance were detected in 69/513 isolates (13.5%). Mutations in *Rv0678*, *Rv1979c* and *mmpL5* associated with CFZ resistance were detected in 79/513 isolates (15.4%). Mutations likely conferring resistance to DLM were detected in *fbiB* and *ddn* genes for 11/513 isolates (2.1%). A mutation associated with PA-824 resistance was detected in the *ddn* gene for 3/513 isolate (0.6%). Nineteen mutations of *pykA, cycA, ald* and *alr* genes associated with CS resistance were found in 153/513 isolates (29.8%). No known mutations associated with LZD were detected (Table 1).

**Table 1.** Proportion of phenotypically XDR isolates (n = 513) with potential resistance-conferring mutations against six drugs.

Drugs	Gene	Locus	Amino acid change	Nucleotide change	Genomic position	No. of strains found, n = 513	DR proportion, n = 513
BDQ	*Rv0678*	Rv0678	Leu117Arg	T350G	779339	1 (0.2%)	69/513 (13.5%)
	*Rv0678*	Rv0678	Met146Thr	T437C	779426	1 (0.2%)	
	*Rv1979c*	Rv1979c	Met245Leu	A733C	2222432	2 (0.4%)	
	*Rv1979c*	Rv1979c	Arg409Gln	G1226A	2221939	65 (12.7%)	
CFZ	*Rv0678*	Rv0678	Met146Thr	T437C	779426	1 (0.2%)	79/513 (15.4%)
	*Rv0678*	Rv0678	Leu117Arg	T350G	779339	1 (0.2%)	
	*Rv1979c*	Rv1979c	Arg409Gln	G1226A	2221939	65 (12.7%)	
	*Rv1979c*	Rv1979c	Asp286Gly	A857G	2222308	12 (2.3%)	
	*mmpL5*	Rv0676c	Phe696Leu	T2086C	776395	6 (1.2%)	
DLM	*fbiB*	Rv3262	Lys448Arg	A1343G	3642877	7 (1.4%)	11/513 (2.1%)
	*fbiB*	Rv3262	Leu447Arg	T1340G	3642874	1 (0.2%)	
	*ddn*	Rv3547	Gly81Ser	G241A	3987084	3 (0.6%)	
PA-824	*ddn*	Rv3547	Gly81Ser	G241A	3987084	3 (0.6%)	3/513 (0.6%)
CS	*pykA upstream*	Rv1617 upstream	Gene upstream	C-58T	1816131	2 (0.4%)	153/513 (29.8%)
	*cycA*	Rv1704c	Synonymous SNP	T1050C	1930407	22 (4.3%)	
	*cycA*	Rv1704c	Arg477Gly	C1429G	1930028	15 (2.9%)	
	*cycA*	Rv1704c	Synonymous SNP	C238T	1931219	15 (2.9%)	
	*ald*	Rv2780	Glu118Ala	A353C	3087172	1 (0.2%)	
	*alr*	Rv3423c	Ser22Leu	C65T	3841356	3 (0.6%)	
	*alr*	Rv3423c	Leu113Arg	T338G	3841083	38 (7.4%)	
	*alr*	Rv3423c	Met343Thr	T1028C	3840393	11 (2.1%)	
	*alr*	Rv3423c	Tyr388Asp	T1162G	3840259	5 (1.0%)	
	*alr*	Rv3423c	Synonymous SNP	C52A	3841369	1 (0.2%)	
	*alr upstream*	Rv3423c upstream	Gene upstream	G-243A	3841663	67 (13.1%)	
	*ald*	Rv2780	Val369fs	1106_1107 insG	3087926	1 (0.2%)	
	*ald*	Rv2780	Ser43fs	128_129 insG	3086948	1 (0.2%)	
	*ald*	Rv2780	Gly145fs	433_434 insGC	3087253	3 (0.6%)	
	*ald*	Rv2780	Gly154fs	460_del	3087279	6 (1.2%)	
	*ald*	Rv2780	Thr293fs	877_878 insCG	3087697	1 (0.2%)	
	*ald*	Rv2780	Ala299fs	896_897 insGA	3087716	1 (0.2%)	
	*ald*	Rv2780	Glu323fs	966_967 insGA	3087786	2 (0.4%)	
	*alr*	Rv3423c	Phe4Leu	T10C	3841411	2 (0.4%)	
LZD	*–*	–	–	–	–	0 (0.0%)	0/513 (0.0%)

Note: ins, insertion; del, deletion; fs, frameshift; BDQ, bedaquiline; LZD, linezolid; CFZ, clofazimine; DLM, delamanid; PA or PA-824, pretomanid; CS, cycloserine.

### Lineage classification of XDR-TB strains

**Figure 2.**
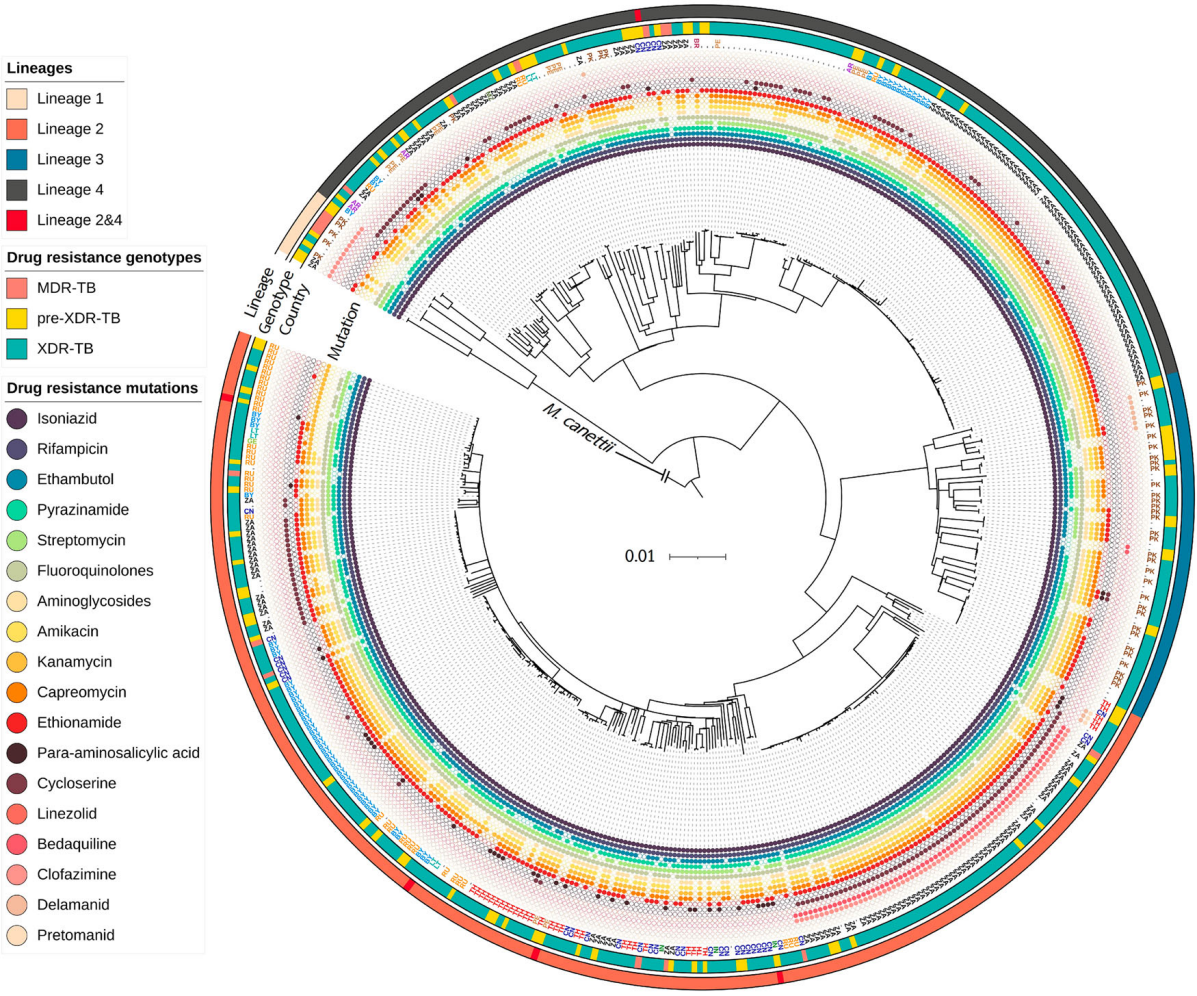
Maximum-likelihood phylogenetic tree for 513 phenotypically XDR isolates. We used *M. canettii* as an outgroup. From inner to outer rings: drug-resistance mutations, country or origin, WGS-based genotypic drug-resistant profile (MDR-TB, pre-XDR-TB, and XDR-TB) and lineages. The genetic distance proportional to the total number of single nucleotide polymorphisms (SNPs) is indicated by the scale bar. TB, tuberculosis; MDR, multidrug resistance; XDR, extensively drug-resistance.

Of the 513 isolates, 250 (48.7%) belonged to lineage 2 (East-Asian lineage), 185 (36.1%) to lineage 4 (Euro-American lineage), 61 (11.9%) to lineage 3 (East African-Indian lineage) and 12 (2.3%) to lineage 1 (Indo-Oceanic lineage). Some isolates belonged to both lineage 2 and lineage 4 (n = 5; 1.0%) (Figure 2 and Supplementary File 3: Table S3). The main sub-lineage of lineage 2 was 2.2.1 (n = 177; 34.5%).

### Cluster analysis and possible transmission clusters

Phylogenetic structure of the 513 phenotypically XDR-TB strains is shown in Figure 2. On average, pairs of isolates differed by 499 ± 251 (mean ± SD) SNPs. In total, 408 isolates (79.5%) fell into 99 clusters (isolates differing by ≤11 SNPs) which each contained between 2 and 21 isolates (Supplementary File 4: Table S4). The proportion of clusters in each DR-TB type (based on genotypic DST) were 29.4% (5/17) for MDR-TB, 74.5% (70/94) for pre-XDR-TB, and 82.8% (333/402) for XDR-TB. The pairwise SNP distances among the 99 clusters are shown in Supplementary File 5: Table S5, where pairwise differences within each cluster are enclosed in shaded boxes.

**Figure 3.**
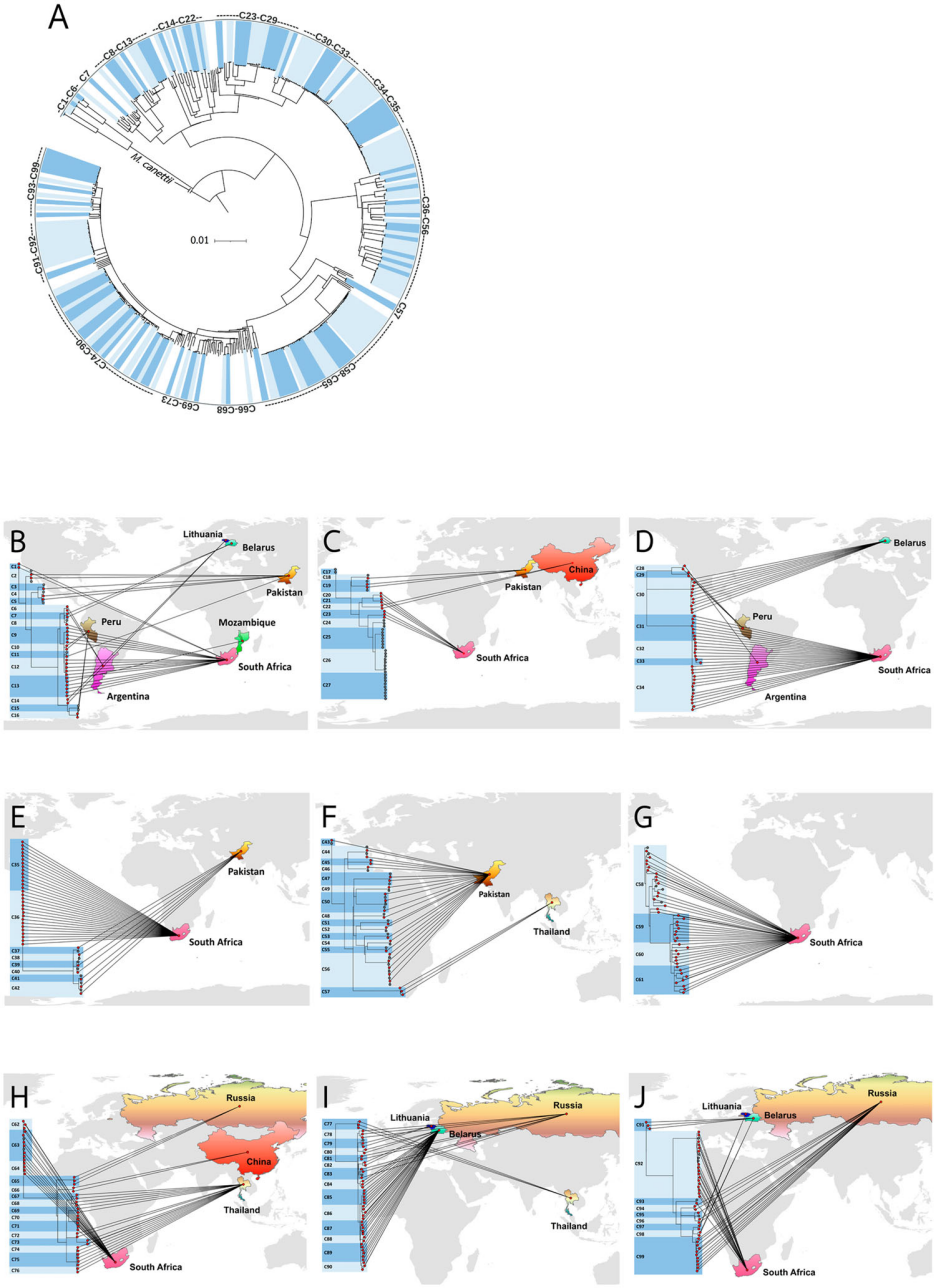
Geographical distribution of clustered drug-resistant isolates in this study. (A) The 99 clusters are labelled in alternating dark and light blue segments in the outer ring. The scale bar shows genetic distance proportional to the total number of SNPs. (B–J) 9 phylo-maps showing geographic links of each cluster to its country of origin.

Most isolates within a cluster shared the same country of origin. 26/99 clusters (26.3%) came from Pakistan. In South Africa, 24/99 clusters (24.2%) were shared. Some clusters were found in more than one country; C13 (Mozambique and South Africa) and C28 (Argentina and Peru) (Figure 3, (A–J); Supplementary File 4: Table S4).

Among the 408 clustered isolates, 354 (86.8%) were identified as possible primary DR-TB. The remaining (13.2%; n = 54/408) were classified as possible acquired DR-TB (Table 2 and Supplementary File 6: Table S6).

**Table 2.** Classification of primary versus acquired resistance of 408 phenotypic XDR-TB isolates among 99 clusters.

Clustered isolates, n = 408	DR-TB types based on genotypic DST, no. (%)		
	MDR-TB, n = 5	pre-XDR-TB, n = 70	XDR-TB, n = 333
Possible primary DR-TB, n = 354, 86.8%	5 (100.0%)	57 (81.4%)	292 (87.7%)
Possible acquired DR-TB, n = 54, 13.2%	0 (0.0%)	13 (18.6%)	41 (12.3%)

### Drug susceptibility results associated with clustered isolates and possible DR-TB transmission clusters

Isolates that fell within clusters were significantly (*p* values < .05) associated with phenotypic resistance against pyrazinamide (Z) and KM and genotypic resistance against Z, ethambutol (E), FQ, aminoglycosides (AMG), AM, KM, CM, ethionamide (ETO), para-aminosalicylic acid (PAS), CS, BDQ, and CFZ. The proportion of genotypically PAS-resistant strains was significantly higher among non-clustered isolates (Supplementary file 7 Table S7). Possible primary DR-TB transmission clusters were significantly (*p* values < .05) associated with genotypic resistance against AMG, AM, CM, and ETO (Supplementary file 8 Table S8).

## Discussion

XDR-TB is a serious problem. Neither the predicted efficacy of the newly recommended anti-TB drugs for treating DR-TB nor the proportion of primary versus acquired resistance cases among global XDR-TB cases have been clearly reported. In this study, we have estimated the efficacy of these drugs and found examples of primary XDR-TB globally.

In 2020, WHO revised its recommendations for drugs to use against DR-TB [2]. Given their high bactericidal activities, these drugs are expected to be very effective for treatment of MDR and XDR-TB cases [29]. However, the extent to which isolates already exhibiting resistance to many current drugs exhibit resistance to the newly recommended drugs remains unknown globally. Therefore, we investigated genotypic resistance based on publicly available WGS data for DR-TB isolates. We assumed that resistance against new drugs should be more common in M/XDR-TB strains because of their exposure to a range of drugs during treatment. Therefore, investigation of M/XDR-TB strains would provide an initial estimation of the effectiveness of the newly recommended drugs. It is difficult to collect sufficient XDR samples to test phenotypically, so we opted for genotypic DSTs using WGS data. We assembled a database of known mutations retrieved from analysis tools and the primary literature. Our screening found a low proportion of phenotypically XDR-TB strains that were genotypically resistant to DLM and PA-824: fewer than 3% of the tested strains had mutations associated with resistance to these drugs. For BDQ and CFZ, we found a higher proportion of resistant strains (13.5% and 15.4%, respectively).

A previous study reported that only 2% (n = 22/1,085) of randomly selected *Mtb* isolates, collected between 1991 and 2018 across 114 countries, contained a mutation in BDQ resistance-candidate genes (*atpE*, *mmpR5* and *pepQ*) [30]. In our study, the proportion of potentially BDQ-resistant isolates was relatively high (13.5). This may be due to the fact that we used phenotypically XDR-TB strains. Also, many of the WGS datasets we used were from a single country, South Africa (n = 181/513: 35.3%). Concordantly, most isolates resistant to BDQ were also from South Africa (n = 58/69: 84.1%). This is one limitation of our study: we retrieved only publicly available WGS datasets of XDR-TB strains from particular countries and BDQ resistance has the highest prevalence in South Africa. Note that WHO recommended in 2020 that MDR-TB, with or without fluoroquinolone resistance, should be treated using oral regimens that include BDQ [2]. The high prevalence of BDQ resistance, at least in South Africa, could therefore greatly affect treatment efficacy against DR-TB.

There is potential for BDQ and CFZ cross-resistance due to shared mutations in *Rv0678* and *Rv1979c* genes [31]. In our study, we found *Rv0678* [T350G (Leu117Arg) and T437C (Met146Thr)] and *Rv1979c* [G1226A (Arg409Gln)] in the strains resistant to both BDQ and CFZ, supporting the likelihood of cross-resistance. An LZD resistance-associated mutation, *rrl* (C344T) (WHO group 5; not associated with DR [32]) was found in low proportions (0.6%) (Supplementary File 1: Table S1) and we also identified it as a neutral mutation since this mutation was found in more than 5% of pan-susceptible strains that we studied. Although *rplC* [T460C (Cys154Arg)] is an established marker for LZD resistance (WHO group 1), and was previously reported to be the most prevalent mutation in phenotypically LZD-resistant isolates among M/XDR-TB strains in China [33], none of the XDR-TB strains that we studied contained this mutation. In the case of DLM, we detected a low proportion of resistant strains (2.1%). Associated mutations were in the *fbiB* and *ddn* genes, the majority in *fbiB*. Normally, DLM resistance is driven by mutations in the *ddn*, *fbiA*, *fbiB*, *fbiC* and *fgd1* genes, since DLM inhibits production of mycolic acid and needs stimulation by the F420-dependent nitroreductase produced by the *ddn* gene. The enzymes encoded by the *fbiA*, *fbiB*, *fbiC* and *fgd1* genes produce the F420 cofactor. Polymorphisms in these genes are linked to phenotypic DLM resistance [34,35]. A recent study has reported a novel mutation in *fbiD* (Rv2983), a gene that encodes a guanylyltransferase required for cofactor F420 biosynthesis, as a novel determinant of DLM and PA-824 resistance in *Mtb* [36]. Nonetheless, we did not detect any strains with *fbiD* mutations that might indicate DLM and PA-824 resistance. The low prevalence we noted in our XDR-TB strains of mutations conferring resistance to BDQ, CFZ, LZD, DLM and PA-824 suggests that these drugs could have high efficacy for treatment of XDR-TB.

More than 29% of the XDR isolates we studied were resistant to CS, possibly as a consequence of the long usage of this drug for TB treatment. Despite our efforts to include all known CS resistance-associated genes, (22 genes with 63 mutations), we found DR mutations only in the *pykA* gene (encoding pyruvate kinase), *cycA* gene (encoding D-serine/alanine/glycine transporter protein), *ald* gene (encoding alanine dehydrogenase) and *alr* gene (encoding alanine racemase). A previous study reported novel mutations in various genes associated with CS resistance and in genes involved in variety of biological mechanisms such as stress response, lipid metabolism, methyltransferase, and transport systems [37]. Few CS resistance-associated genes were found in XDR-TB strains in our study. As CS and CFZ belong to the same group (Group B) of WHO-recommended drugs for DR-TB treatment [2], CFZ might be a better choice to include in the regimen, especially in the XDR-TB treatment.

Here, we reported exact matches to known resistance-conferring mutations. However, some other variants that may occur at the same sites were not considered as DR-associated mutations. For example, whereas deletion of a codon from *fbiD* [385_delA, 386_delT, and 387_delC (Ile129_del)] is regarded as resistance-conferring against DLM and PA-824, we found only a single-nucleotide deletion [387_delC (Ile129fs)] (Supplementary File 1: Table S1). To balance between the sensitivity and specificity of genotypic resistance to these six important drugs, we included several mutations from previous studies, but also excluded low-confidence variants.

To understand XDR-TB transmission, we analysed the clustering of related strains on a global scale. Acquisition of drug resistance by TB is commonly driven by low compliance of the patient in taking anti-TB drugs [38]. Alternatively, the transmission of pre-existing DR-TB leads to primary DR-TB. However, the relative proportions and magnitudes of primary and acquired resistance at the global level have never reported. Previously, our group has studied the transmission of MDR/XDR-TB at the nation-wide level in Thailand and identified the majority of clustering isolates (82%) as primary DR-TB. More than three-quarters of MDR-TB and XDR-TB isolates and two thirds of pre-XDR-TB isolates were regarded as examples of primary resistance [7]. Here, we further demonstrate likely transmission events of XDR-TB in a global setting.

In some regions of South Africa, there is evidence that DR-TB spreads by clonal expansion and transmission of a single strain with multiple resistance mutations [39]. WGS investigation of a large XDR-TB cohort (>400 cases) from a single province in South Africa revealed that only 30% of patients had established epidemiologic linkages (person-to-person or hospital links) and 70% of transmission events might have come from casual contact between people who did not know each other [40]. In Panama, WGS analysis of 66 MDR isolates, revealed that 78% belonged to the Latin American-Mediterranean (LAM) family, and that there was a high proportion of clustering isolates (43.9%, 29/66 isolates). Alternatively, there are also studies from South Africa reporting that DR-TB arises by acquired resistance (repeated selection for resistance) rather than by clonally derived transmission [41,42]. The issue of whether DR-TB spreads as primary resistance or is acquired resistance in the patient remains unclear.

In our phylogenetic tree, using a SNP distance cut-off value of ≤11 SNPs, we found 99 clusters of DR-TB among the global XDR-TB samples. Members in most clusters shared their country of origin. The largest clusters contained 21 isolates (C58 and C92) that were mostly found in South Africa. Based on our analysis of the acquisition of additional resistance-associated mutations, we identified likely primary resistance in 86.8% (354/408 isolates) of all clustered isolates, including 100% of MDR-TB, 81.4% of pre-XDR-TB, and 87.7% of XDR-TB cases. Twenty-five clusters contained isolates with mixed DR types based on genotypic susceptibility testing. Based on the available data of XDR-TB from many countries, primary XDR-TB is more prevalent in transmission events than is acquired resistance, except in Argentina. Overall, more than 80% of the phenotypically XDR-TB strains derived from possible transmission events of primary XDR-TB in each region. So, primary XDR-TB transmission could be the key contributor to XDR-TB prevalence worldwide. This finding could advance the knowledge on XDR-TB transmission and support appropriate control methods around the world. We identified clusters using a highly stringent SNP threshold (≤11 SNPs) based on 17,988 high-confidence SNPs*.* Our use of such a criterion to identify clusters arose because we needed to ensure that the M/XDR-TB clusters were not incorrectly inferred due to use of a SNP difference threshold that was too high. Nevertheless, we found that some isolates in small clusters should be combined with larger clusters due to the cross correlation between clusters based on SNP distance and co-ancestral relationships (e.g. clusters C35 and C36 (Figure 3)).

DST (especially genotypic) results were strongly associated with clustered isolates and possible DR-TB transmission clusters. Most isolates falling into clusters had the ability to resist anti-TB drugs, indicating that the fitness cost due to drug resistance [43] does not affect the transmissibility of DR-TB. We also found that genotypically PAS-resistant strains were significantly over-represented in non-clustered isolates. So, non-spreading XDR-TB, which is caused by acquired resistance, is more commonly associated with PAS resistance, suggesting that the mechanism of PAS resistance is more related to low compliance of taking anti-TB drugs than transmission. Clustered primary DR-TB isolates were significantly associated with AMG, AM, CM, and ETO resistance compared to non-spreading XDR-TB (Supplementary file 8 Table S8). Thus, there is a greater chance of resistance to these four drugs in primary XDR-TB patients. Alternatively, these four drugs could be a better choice in treating patients with acquired DR-TB.

Our study has limitations that should be noted. To compensate for the lack of phenotypic DST results for the six drugs of interest, known resistance-conferring mutations were retrieved from various mutation databases and publications (Supplementary File 1: Table S1). The strains that we recruited were regarded as XDR based on the previous definition of XDR-TB, as this was the definition used at the time, and formed the basis of treatment decisions [16]. Nonetheless, the estimation of resistance against the six newly recommended drugs is still valid. Further studies are needed to verify our findings in clinical phenotypically XDR-TB isolates. We assumed possible transmission cluster events only based on the acquisition of additional resistance-related mutations and the genetic distances among the isolates. There were no fine-scale data for epidemiologic links, no data for exposure and treatment histories of the cases to identify the index cases and differentiate between primary and acquired resistance. The transmission levels of XDR-TB strains, as per the new definition [1], are therefore still unknown. As the drugs we researched are still not routinely used in many countries, we assumed that the transmission of strains classified under the new definition of XDR-TB might be not high. Alternatively, based on our data, it might be that transmission of XDR-TB based on the new definition could be high in the future unless the proper intervention for stopping the transmission chain has been introduced.

In conclusion, among 513 phenotypically XDR-TB isolates, we found low proportions of mutations conferring resistance to five of the newly recommended drugs (BDQ, CFZ, LZD, DLM and PA-824). There seems to be a higher proportion of DR to CS, the sixth recommended drug, than to the other drugs. No isolates were genotypically resistant to LZD. We also showed that most M/XDR-TB cases around the world might be caused by clonal transmission of primary M/XDR-TB, which might go a long way towards explaining the high global prevalence of DR-TB. Moreover, we found a significant association between DST profile, possible primary XDR-TB, and clustering of isolates. These findings may be used to improve XDR-TB treatment and control.

## Supplementary Material

Supplemental Material
